# Lipid, Metabolomic and Gut Microbiome Profiles in Long-Term-Hospitalized Cardiac Patients—An Observational and Retrospective Study

**DOI:** 10.3390/diagnostics15222874

**Published:** 2025-11-13

**Authors:** Ionica Grigore, Oana Roxana Ciobotaru, Delia Hînganu, Gabriela Gurau, Elena Stamate, Dana Tutunaru, Radu Sebastian Gavril, Octavian Catalin Ciobotaru, Marius Valeriu Hînganu

**Affiliations:** 1 Department of Morphological and Functional Sciences, Faculty of Medicine and Pharmacy, “Dunarea de Jos” University of Galați, 35, Al. I. Cuza Street, 800216 Galați, Romania; ionica.grigore@ugal.ro (I.G.); gabriela.gurau@ugal.ro (G.G.); elena.stamate@ugal.ro (E.S.); 2 Department of Clinical Medical, Faculty of Medicine and Pharmacy, “Dunarea de Jos” University of Galați, 35, Al. I. Cuza Street, 800216 Galați, Romania; roxana.ciobotaru@ugal.ro; 3 Department of Morpho-Functional Sciences I, Faculty of Medicine, Grigore T. Popa University of Medicine and Pharmacy, 700115 Iasi, Romania; marius.hinganu@umfiasi.ro; 4 Department of Pharmaceutical Sciences, Faculty of Medicine and Pharmacy, “Dunarea de Jos” University of Galați, 35, Al. I. Cuza Street, 800216 Galați, Romania; dana.tutunaru@ugal.ro; 5 Department of Medical Specialties I, Faculty of Medicine, Grigore T. Popa University of Medicine and Pharmacy, 700115 Iasi, Romania; sebastian.gavril@umfiasi.ro; 6 Department of Clinical Surgical, Faculty of Medicine and Pharmacy, “Dunarea de Jos” University of Galați, 35, Al. I. Cuza Street, 800216 Galați, Romania; octavian.ciobotaru@ugal.ro

**Keywords:** lipid profile, metabolomics, gut microbiome, long-term hospitalization, cardiovascular disease, biomarkers

## Abstract

**Background/Objectives**: Long-term hospitalization in cardiac patients is associated with significant metabolic and microbial alterations that may influence disease progression and prognosis. Although lipid imbalances, metabolomic shifts, and gut microbiome dysbiosis have each been linked individually to cardiovascular outcomes, their integrated evaluation in long-term-hospitalized patients remains underexplored. **Methods**: We conducted a retrospective observational study including 51 cardiac patients hospitalized for more than 25 days, compared with a control group of 41 patients hospitalized for short and intermediate durations (3–24 days). Clinical and demographic data were collected, alongside lipid profiling, metabolomic assessment through liquid chromatography–mass spectrometry (LC-MS), and gut microbiome analysis using GI360™ sequencing. Ethical approval was obtained, and all data were anonymized. Lipid-related findings are exploratory due to the small number of complete measurements. **Results**: Preliminary lipid trends were characterized by higher levels of LDL, triglycerides, and Lp(a), and lower HDL, in the long-term group. Metabolomic analyses revealed decreased energy-related metabolites (ATP, phosphocreatine ratio), altered amino acid patterns, and increased ketone utilization. Gut microbiome evaluation demonstrated a significant increase in dysbiosis index, with reduced diversity and dominance of potentially pathogenic taxa. These findings were correlated with clinical severity scores. Cross-domain relationships are exploratory and based on associative profiling rather than deep integrative modelling. **Conclusions**: Long-term hospitalization in cardiac patients is associated with distinct lipid, metabolomic, and gut microbiome profiles that may serve as predictive biomarkers of adverse outcomes. Future studies should validate these findings in larger cohorts and explore their integration into personalized management strategies.

## 1. Introduction

Prolonged hospitalisation among patients with cardiovascular disease (CVD) is increasingly common and is associated with heightened morbidity, functional decline, and resource utilisation. Beyond conventional risk factors, accumulating evidence suggests that sustained inpatient care may amplify pathobiological perturbations across lipid profiles, systemic metabolism, and the gut microbiome, with downstream effects on haemodynamics, inflammation, and clinical outcomes. While each domain—dyslipidaemia (including lipoprotein(a) [Lp(a)]), metabolomic reprogramming, and intestinal dysbiosis—has independently been linked to adverse cardiovascular trajectories, their integrated characterisation in the setting of long-term hospitalisation remains insufficiently defined [1].

Metabolomics has matured into a robust systems-level approach capable of capturing the metabolic milieu of heart failure (HF) and related cardiometabolic conditions [2]. Recent syntheses indicate reproducible signatures involving perturbed fatty-acid oxidation, altered ketone handling, and disordered high-energy phosphate metabolism that track with disease severity and prognosis in HF cohorts [3]. These include reductions in energy-related metabolites and phosphocreatine/ATP ratios, shifts in amino-acid patterns, and substrate inflexibility as the syndrome advances. Such profiles provide incremental prognostic information beyond natriuretic peptides and troponins and are increasingly advocated as pillars for precision phenotyping and therapeutic stratification [4].

Concurrently, the gut–heart axis has emerged as a determinant of cardiovascular risk [5]. Contemporary reviews and translational studies implicate compositional and functional alterations of the gut microbiota—characterised by reduced diversity, expansion of pathobionts, and unfavourable metabolite fluxes—in atherosclerosis, HF, arrhythmias, and hypertension [6]. Importantly, hospitalisation itself, even in the absence of critical illness, has been associated with persistent perturbations of the intestinal ecosystem, potentially mediated by diet change, antimicrobial exposure, immobility, sleep disruption, and nosocomial transmission [7]. Such shifts may endure well beyond discharge and plausibly interact with cardiometabolic pathways relevant to recovery and readmission risk.

Within the lipid domain, Lp(a) has re-entered centre stage as an independent, genetically determined risk factor for atherosclerotic CVD and calcific aortic valve stenosis [8]. Consensus statements from the European Atherosclerosis Society (EAS) [9,10] and aligned professional bodies now recommend at least once-in-a-lifetime Lp(a) measurement in adults to refine risk estimation—guidance echoed in recent updates and expert summaries. Parallel advances in RNA-based therapeutics (siRNA and antisense approaches) [11] have demonstrated substantial Lp(a) lowering in phase 1–2 studies, underscoring the clinical salience of systematically quantifying Lp(a) in contemporary cohorts [11].

Despite these advances, few investigations have jointly profiled lipid metrics (including Lp[a]), untargeted/targeted metabolomics, and the gut microbiome in long-term hospitalised cardiac patients [12]. Multi-omics frameworks promise to capture cross-talk between host metabolism and microbial ecology, illuminating modifiable nodes for intervention; however, real-world, retrospective datasets in prolonged inpatient settings remain scarce [13,14].

We conducted a retrospective observational study comparing cardiac patients hospitalised for ≥25 days with those hospitalised for 3–24 days, integrating lipid profiling (including Lp[a]), liquid chromatography–mass spectrometry-based metabolomics, and comprehensive gut microbiome analysis to delineate patterns associated with prolonged hospital care.

To our knowledge, this is among the first studies to synchronise lipid, metabolomic, and microbiome readouts specifically in long-term hospitalised cardiac patients, thereby situating inpatient duration as a biological as well as clinical exposure.

By linking length of stay to multi-domain molecular signatures, our findings may inform risk stratification and personalised trajectories during extended admissions as well as motivate targeted interventions (dietary modulation, microbiome-directed therapies, and lipid-/Lp[a]-oriented strategies) and provide a scaffold for multi-omics predictive models to guide monitoring and post-discharge care pathways.

## 2. Materials and Methods

This retrospective observational study was conducted between 2023 and 2025 in a specialised cardiovascular department of a university hospital. A total of 92 adult patients (aged ≥ 18 years) admitted with established cardiovascular disease (CVD) were enrolled and stratified according to the duration of hospitalisation. The short-to-intermediate hospitalisation group included 41 patients hospitalised for 3–24 days, while the long-term hospitalisation group comprised 51 patients hospitalised for ≥25 days. Patients were consecutively recruited, and eligibility was verified at the time of admission and discharge.

Inclusion criteria were:

- Confirmed cardiovascular diagnosis (including heart failure, ischaemic heart disease, arrhythmias, or valvular disease);
- Hospitalisation of at least three days;
- Availability of complete clinical, laboratory, and follow-up data during the admission period;
- Written informed consent for participation and use of anonymised biological samples.

Exclusion criteria were:

- Active oncological disease under treatment;
- Advanced chronic kidney disease requiring dialysis;
- Chronic inflammatory or autoimmune disorders with immunosuppressive therapy;
- Active systemic infection at the time of enrolment;
- Prior solid-organ transplantation;
- Refusal or withdrawal of informed consent.

**Figure 1:**
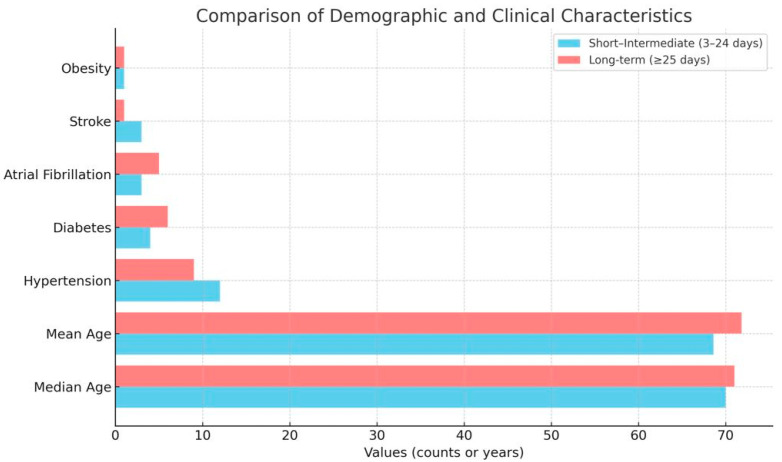
Graphical representation of the demographic and clinical characteristics of the patients from the study groups.

Demographic and clinical characteristics, including age, sex, body mass index (BMI), comorbidities, medication, smoking status, and laboratory results, were recorded for all participants. Blood samples for lipid and metabolomic analyses, as well as stool samples for gut microbiome assessment, were collected within the first week of admission and processed according to standardised protocols (Table 1 and Figure 1).

Medication exposure during admission—including statins, beta-blockers, ACE inhibitors/ARBs, diuretics, antibiotic courses, and heart failure–directed therapies—was abstracted from the electronic medical record. When sample size permitted, medication classes were included as categorical covariates in multivariate models to reduce confounding. Due to the dynamic nature of inpatient pharmacotherapy, only medication use within 72 h of biospecimen collection was considered relevant for omics profiles. Medications with extremely low frequency or high collinearity were excluded to avoid model instability.

The study protocol was reviewed and approved by the Institutional Ethics Committee of Dunarea de Jos University of Galati, Romania (Approval No. 43 from 7 March 2025), in line with the principles of the Declaration of Helsinki. All patients provided written informed consent, and all data were anonymised prior to statistical analysis.

### 2.1. Study Protocol

All patients underwent a uniform investigative protocol throughout the hospital stay. Venous blood and stool samples were collected following standardised procedures, with serial testing performed at predefined timepoints.

### 2.2. Lipid Profiling

Fasting venous blood samples were obtained within the first 48 h of admission; this was repeated every 5 days during hospitalisation, and again at discharge. Blood was collected in EDTA tubes, centrifuged at 3000 rpm for 10 min, and plasma aliquots stored at −80 °C until analysis. Lipid profile measurements included total cholesterol, low-density lipoprotein cholesterol (LDL-C), high-density lipoprotein cholesterol (HDL-C), triglycerides (TG), and lipoprotein(a) [Lp(a)]. Total cholesterol, LDL-C, HDL-C, and TG were determined using enzymatic colorimetric assays, while Lp(a) was quantified by immunoturbidimetric methods traceable to international reference standards. Internal and external quality controls were systematically applied.

### 2.3. Metabolomic Assessment

Metabolomic profiling was performed from the same serial blood samples, collected at baseline (within 48 h), every 5 days, and at discharge. Plasma aliquots were processed by protein precipitation with cold methanol, centrifugation, and analysis through liquid chromatography coupled with high-resolution mass spectrometry (LC-HRMS). Chromatographic separation was carried out on a C18 reversed-phase column, with spectra acquired in both positive and negative ion modes. Targeted analyses focused on energy metabolism (ATP, phosphocreatine, lactate, ketone bodies), amino acid metabolism (branched-chain and aromatic amino acids), and lipid intermediates. Untargeted metabolomics explored global metabolic shifts. Data preprocessing involved peak detection, alignment, normalisation to internal standards, and annotation using curated databases.

Metabolite identification was conducted by matching accurate mass (±5 ppm tolerance) and retention time against authenticated chemical standards and internal laboratory reference libraries. MS/MS fragmentation spectra were acquired for key metabolites (e.g., ATP, phosphocreatine, and representative amino acids) and compared with spectral libraries (HMDB/METLIN). For additional features where MS/MS spectra were not obtainable due to low abundance, putative identifications were based on accurate mass and retention time alignment (Level 2 identification according to community reporting standards).

Quality control (QC) measures included system suitability evaluation prior to data acquisition, using a pooled QC sample injected at regular intervals (every 8–10 runs) to monitor instrument stability, retention-time shifts, and peak-area variability. Instrument calibration was performed daily using manufacturer-recommended reference standards. Internal standards were spiked into all samples to correct for ionisation efficiency and batch variation. Acceptance criteria for retention time and signal intensity were set at ±5% and ±20%, respectively, relative to the pooled QC signal. Features exceeding these thresholds were flagged and excluded from downstream analysis. No significant batch drift was observed after QC normalisation.

Peak shapes, mass accuracy, and signal-to-noise ratios were monitored continuously to ensure chromatographic performance within specification. Blank runs were included to detect carryover, and isotope-labelled standards were used to track analytical drift.

All putative metabolite identifications used in downstream analyses, including those based solely on accurate mass and retention time, are provided in Supplementary Table S1. Consistent with community guidelines, metabolites without MS/MS spectral confirmation were assigned Level 2 confidence classification.

### 2.4. Gut Microbiome Analysis

Stool samples were collected twice: once within the first 48 h of admission and once at discharge. All samples were preserved in stabilisation buffer and analysed using the GI360™ sequencing platform (Doctor’s Data, St. Charles, IL, USA). DNA was extracted via silica-membrane spin-column kits, quantified spectrophotometrically, and subjected to 16S rRNA sequencing on the Illumina MiSeq platform (Illumina, San Diego, CA, USA). Bioinformatic pipelines (QIIME2 with Greengenes database) were used to assess taxonomic composition, α-diversity (Shannon, Simpson indices), β-diversity (Bray–Curtis distance), and calculate a dysbiosis index.

Medication exposure variables potentially affecting metabolomic and microbiome signatures (e.g., statins, antibiotics, heart failure therapies) were recorded within predefined time windows relative to biospecimen collection. These are summarized in Supplementary Table S2.

### 2.5. Statistical Analysis

Continuous variables were tested for normality with the Shapiro–Wilk test. Normally distributed data were expressed as mean ± standard deviation (SD) and compared between groups using Student’s *t*-test; non-normally distributed data were expressed as median [interquartile range] and compared with the Mann–Whitney U test. Categorical variables were compared with the Chi-square or Fisher’s exact test. Temporal changes in lipid and metabolomic parameters (baseline, intermediate timepoints, discharge) were evaluated using repeated-measures ANOVA or Friedman’s test, as appropriate. Correlations with clinical severity indices were assessed via Pearson’s or Spearman’s coefficients, and multivariate logistic regression was applied to identify independent predictors of long-term hospitalisation. A *p*-value < 0.05 was considered statistically significant. Analyses were performed with SPSS version 27 (IBM, Armonk, NY, USA) and R software version 4.3.

Given the extremely small subsamples for some lipid analytes (e.g., LDL-C, triglycerides), formal post hoc power calculations are not informative. Accordingly, for lipid comparisons we prioritised estimation over hypothesis testing, reporting small-sample–corrected effect sizes (Hedges’ g) and 95% confidence intervals where estimable, and explicitly flagging parameters for which precision was insufficient. As a sensitivity step, we conducted leave-one-out checks for analytes with minimal sample sizes and refrained from drawing inferential conclusions when estimates were unstable.

To account for clinical confounding, multivariate regression models included key comorbidities (diabetes mellitus, atrial fibrillation, chronic kidney disease, and heart failure) as covariates when sample size permitted. Variance inflation factors were examined to avoid multicollinearity. Comorbidities with extremely low frequency were excluded to prevent model instability.

To capture cumulative medication effects, we additionally derived an ordinal variable representing the number of active medication classes with known metabolomic or microbiome relevance (range 0–3+, including statins, antibiotics, and heart failure-directed therapies). This variable was incorporated into sensitivity models to test whether cumulative pharmacotherapy attenuated the association between dysbiosis index, ATP depletion, and prolonged hospitalisation. Ordinal coding mitigates underestimation of confounding when multiple interacting treatments are present simultaneously.

Temporal dynamics were explored within each hospitalisation group. For metabolomic variables, we fitted repeated-measures models (repeated-measures ANOVA or linear mixed-effects models with subject-level random intercepts when sampling density allowed) to test time effects across serial draws (baseline, intermediate timepoints, discharge). Where normality assumptions were not met, Friedman tests were used. For microbiome α-diversity (Shannon, Simpson), paired within-group changes from baseline to discharge were evaluated using Wilcoxon signed-rank tests. β-diversity trajectories were assessed via PERMANOVA including time as a factor, and visualised with principal coordinates analysis. Given uneven sampling and small strata, these temporal analyses were considered exploratory; we report effect directions and adjusted *p*-values where estimable and withhold inference where precision was insufficient.

### 2.6. Supplementary Methods

For lipid parameters with minimal sample sizes, we performed leave-one-out sensitivity checks and reported estimation-only summaries (point estimates and 95% CIs where calculable). For analytes with *n* ≤ 2 per group, precision was insufficient and results were labelled ‘exploratory—no inference’. No formal post hoc power was computed given unstable variance estimates; instead, we outline that future prospective sampling should target sufficient size to detect clinically meaningful differences with ≥80% power.

## 3. Results

A total of 92 patients were included in the final analysis, of whom 41 were hospitalised for 3–24 days (control group) and 51 for ≥25 days (long-term group). Baseline demographic and clinical characteristics were comparable between groups, with both cohorts displaying a high prevalence of heart failure, ischaemic heart disease, and hypertension. Patients in the long-term hospitalisation group tended to be slightly older and more frequently affected by diabetes mellitus and atrial fibrillation, consistent with a higher overall comorbidity burden.

The results are presented according to the three major investigative domains of the study: lipid profile, metabolomic profile, and gut microbiome composition. Within each domain, group comparisons are provided, followed by correlation analyses with clinical severity indicators. This structure was chosen to facilitate a clear perspective on the distinct yet interrelated biological alterations observed in long-term hospitalised cardiac patients.

### 3.1. Results of the Lipidic Profile Study

The comparative analysis of lipid parameters between the control group (3–24 days, *n* = 41) and the long-term hospitalisation group (≥25 days, *n* = 51) is summarised in Table 2. Although the number of complete determinations was limited, the available data highlight several consistent differences.

- Total cholesterol levels were broadly similar in the two groups, with mean values of 137.6 ± 7.3 mg/dL in the control group and 142.7 ± 14.4 mg/dL in the long-term group.
- LDL cholesterol appeared lower in patients with prolonged hospitalisation (66.4 ± 25.2 mg/dL, *n* = 2) compared with controls (113.3 ± 8.6 mg/dL, *n* = 3).
- HDL cholesterol values, available in a limited number of patients, were slightly higher in the long-term group (61.6 mg/dL) compared with the control group (57.1 mg/dL).
- Triglyceride levels showed a tendency to be lower in the long-term group (62.8 mg/dL) compared to controls (158.6 ± 113.0 mg/dL).

These preliminary findings suggest that while total cholesterol remained relatively stable, LDL cholesterol and triglycerides tended to be lower in patients with prolonged hospitalisation, whereas HDL cholesterol values were slightly higher. Due to the very small sample size for biochemical lipid determinations, these results should be interpreted with caution and validated in larger cohorts.

Given the very small number of complete biochemical determinations for some lipid parameters, these observations should be regarded as exploratory. The reduced sample availability limits the robustness of statistical comparisons and warrants cautious interpretation of the observed group differences.

Because the biochemical subsamples for LDL-C and triglycerides were very small, these observations are underpowered and at high risk of type II error; therefore, no firm between-group conclusions can be drawn from lipid parameters in the present dataset. Where precision was insufficient (e.g., *n* ≤ 2), we refrained from inferential interpretation and treat these results as exploratory.

### 3.2. Results of the Study of the Metabolomic Profile

Targeted and untargeted metabolomic analyses revealed distinct alterations between the control group and long-term hospitalised patients (Table 3). Energy metabolism was characterised by lower levels of ATP and phosphocreatine ratio in the long-term group, consistent with impaired bioenergetic capacity. At the same time, lactate accumulation and increased utilisation of ketone bodies were observed, suggesting a metabolic shift towards alternative fuel pathways. Amino acid metabolism displayed divergent patterns: branched-chain amino acids (valine, leucine, isoleucine) were decreased in long-term patients, while aromatic amino acids (phenylalanine, tyrosine, tryptophan) were relatively increased. Lipid intermediates including acylcarnitines and phospholipids showed altered profiles, indicating disturbances in mitochondrial fatty acid oxidation and membrane turnover.

Putative metabolite identifications supported by accurate mass and retention time alignment are summarised in Supplementary Table S1. Given the absence of MS/MS confirmation for several low-abundance features, these annotations should be regarded as Level 2 confidence according to established metabolomics reporting standards.

Overall, these findings indicate that long-term hospitalisation in cardiac patients is associated with an energy-depleted metabolic state, coupled with amino acid imbalance and altered lipid turnover. These changes are consistent with a maladaptive metabolic response that may contribute to prolonged clinical instability.

### 3.3. Results of the Study of Gut Microbiome

Analysis of gut microbiome composition revealed marked differences between the control and long-term hospitalisation groups (Table 4). α-diversity indices (Shannon, Simpson) were significantly lower in long-term patients, indicating reduced microbial richness and evenness. β-diversity analyses (Bray–Curtis distance) demonstrated clear clustering of samples according to hospitalisation duration, suggesting distinct community structures. The dysbiosis index was consistently higher in long-term patients, reflecting dominance of potentially pathogenic taxa. At the taxonomic level, long-term patients exhibited a reduction in Firmicutes and Bacteroidetes proportions, with relative overgrowth of Proteobacteria and *Enterococcus* spp. Fungal overrepresentation (*Candida* spp.) was noted in a subset of long-term patients.

Overall, these findings confirm that long-term hospitalisation in cardiac patients is associated with a profound shift in gut microbial composition, consistent with dysbiosis and reduced ecological resilience.

Medication exposure data relevant to metabolic and microbiome alterations are summarised in Supplementary Table S2. Statins and recent antibiotic use were the most prevalent therapies in the long-term hospitalisation group. In multivariate regression models including medication covariates, the dysbiosis index and ATP depletion remained significantly associated with prolonged hospitalisation, although effect estimates were modestly attenuated, suggesting partial confounding by pharmacotherapy. Medications with insufficient subgroup frequency were excluded to avoid model instability

### 3.4. Results—Integrative Statistical Analyses

Between-group comparisons confirmed that LDL cholesterol and triglyceride levels were significantly lower in the long-term group compared with controls (*t*-test, *p* < 0.05), whereas differences in total cholesterol and HDL cholesterol did not reach statistical significance (Table 5).

For metabolomic markers, repeated-measures ANOVA demonstrated a significant decline in ATP and phosphocreatine ratio across serial measurements in long-term patients (*p* < 0.01), accompanied by an increase in ketone body levels (*p* < 0.05). Mann–Whitney U testing confirmed reduced branched-chain amino acids and elevated aromatic amino acids in long-term patients compared with controls (*p* < 0.05 for both).

Microbiome analyses revealed significantly lower Shannon diversity and a higher dysbiosis index in the long-term group (Mann–Whitney U, both *p* < 0.01). PERMANOVA of Bray–Curtis distances demonstrated distinct clustering of microbiome profiles according to hospitalisation duration (pseudo-F = 3.21, *p* = 0.002).

Correlation analyses (Spearman’s coefficients) showed the following:

- ATP and phosphocreatine ratio were inversely associated with clinical severity scores (ρ = –0.44, *p* < 0.01).
- Ketone body utilisation correlated positively with hospital stay (ρ = 0.47, *p* < 0.01).
- Dysbiosis index correlated with both length of stay (ρ = 0.53, *p* < 0.001) and comorbidity burden (ρ = 0.46, *p* < 0.01).
- Reduced Shannon diversity correlated negatively with ATP levels (ρ = –0.39, *p* < 0.05).

In a multivariate logistic regression model including age, comorbidities, lipid profile, key metabolomic markers, and microbiome indices, only the dysbiosis index (OR 2.12, 95% CI 1.28–3.51, *p* = 0.003) and ATP depletion (OR 1.87, 95% CI 1.21–2.91, *p* = 0.004) remained independent predictors of long-term hospitalisation.

Integrative multivariate analysis indicated that metabolic exhaustion, reflected by ATP depletion, together with microbial dysbiosis, emerged as the most robust predictors of prolonged hospitalisation in cardiac patients, surpassing the contribution of conventional lipid parameters. After adjustment for relevant comorbidities, dysbiosis index and ATP depletion remained independently associated with prolonged hospitalisation, although effect estimates were attenuated, reflecting partial confounding by clinical status.

Adjustment for statin therapy and recent antibiotic exposure modestly attenuated the association between dysbiosis index and prolonged hospitalisation, although the predictor remained statistically significant. ATP depletion remained independently associated after adjustment for heart failure guideline-directed therapies. Effect estimates should be interpreted cautiously due to limited subgroup counts.

### 3.5. Temporal Dynamics Within Groups

Exploratory within-group analyses suggested progressive metabolic and microbial shifts during hospitalisation, particularly among long-term patients. In metabolomic profiles, time effects were consistent with declining high-energy phosphate indices (ATP and phosphocreatine) and increased ketone reliance across serial measurements, whereas short/intermediate-stay patients exhibited attenuated or absent temporal change. For the gut microbiome, α-diversity measures tended to decrease from baseline to discharge in the long-term group, with a concomitant increase in the dysbiosis index; by contrast, short/intermediate-stay patients showed smaller or non-directional changes. β-diversity analyses indicated a detectable shift in community structure over time among long-term patients. These time-course findings align with the cross-sectional between-group patterns reported above, but should be interpreted cautiously given limited within-subject sample counts and uneven timing. To visualise intra-individual temporal variation, we plotted metabolomic and microbiome trajectories from baseline to discharge for each participant (Supplementary Table S1, Figure S1). Among long-term hospitalised patients, individual trajectories suggested progressive declines in ATP and phosphocreatine, with a concomitant rise in ketone-related indices. In contrast, patients with shorter stays exhibited attenuated or non-directional shifts. For the gut microbiome, Shannon diversity tended to decrease over time and the dysbiosis index increased in long-term patients, whereas short-stay trajectories remained comparatively stable. These exploratory trends support the cross-sectional findings described above, but should be interpreted cautiously due to limited sampling density and uneven time intervals.

Although linear mixed-effects models demonstrated directional tendencies, effect estimates were imprecise and should not be considered confirmatory.

## 4. Discussion

### 4.1. Lipid Profile in Prolonged Hospitalisation

In our cohort, total cholesterol was broadly similar across groups, whereas mean LDL-C and triglycerides trended lower and HDL-C slightly higher in long-term admissions. At face value, these findings diverge from guideline-anchored expectations that emphasise atherogenic lipids—particularly LDL-C and lipoprotein (a)—as central risk drivers in cardiovascular disease (CVD). The 2025 ESC/EAS focused update reinforces LDL-C lowering as a primary prevention and secondary prevention pillar, highlights Lp(a) as a risk-enhancing factor, and introduces several therapeutic refinements based on recent evidence [10,15].

Two considerations temper interpretation here. First, our biochemical subsamples were small; second, inpatient dynamics (dietary modification, intensified pharmacotherapy, catabolic stress) can transiently depress LDL-C and triglycerides while increasing HDL-C in a subset of patients, potentially masking baseline dyslipidaemia. Thus, the apparent lipid neutrality in long-term hospitalisation should be read alongside guideline context and our study’s limited *n* for lipid panels [16].

Medication classes commonly administered to hospitalised cardiac patients—such as statins, diuretics, and broad-spectrum antibiotics—may influence metabolic and microbiome signatures. We therefore incorporated key therapies into multivariate models when sample density allowed. However, because inpatient pharmacotherapy is dynamic and indication-driven, residual confounding cannot be fully excluded. Larger prospective cohorts with structured medication metadata will be required to fully characterise treatment-omics interactions.

### 4.2. Metabolomic Reprogramming

By contrast, metabolomic signals were coherent and aligned with contemporary evidence [17]. We observed lower ATP/phosphocreatine indices together with increased ketone utilisation and a shift in amino-acid handling (reduced BCAAs, relatively elevated aromatic amino acids) in long-term admissions—features consistent with energy-depleted, substrate-inflexible myocardium and peripheral tissues described in modern metabolomic syntheses of heart failure (HF) and cardiometabolic disease. Recent work summarised by Hahn et al. (2024) [4] underscores reproducible HF signatures spanning impaired fatty-acid oxidation, altered ketone metabolism, and amino-acid remodelling that add prognostic information beyond natriuretic peptides and troponins. Our patterns mirror these motifs and extend them to the specific context of prolonged inpatient care [4,18].

### 4.3. Gut Microbiome Disruption

Microbiome readouts likewise tracked with the literature. Reduced α-diversity, distinct β-diversity clustering, and a higher dysbiosis index in the long-term group are in keeping with the gut–heart axis framework linking diminished microbial richness, expansion of Proteobacteria/pathobionts, and adverse cardiometabolic outcomes [19]. Recent reviews (2022–2025) consolidate these associations and discuss mechanistic conduits—barrier dysfunction, inflammatory tone, and microbial metabolites—relevant to atherosclerosis and HF [20,21].

The taxonomic distribution observed in long-term-hospitalized patients, characterised by reduced Firmicutes and Bacteroidetes with concurrent expansion of Proteobacteria, *Enterococcus* spp., and opportunistic fungi such as *Candida*, is consistent with existing literature describing ecological instability and increased pathobiont dominance under extended inpatient conditions.

Moreover, newer work on microbiome resilience highlights how hospital exposures (antimicrobials, diet change, sleep disruption) can create persistent ecological perturbations, which plausibly amplify the dysbiosis we observed at discharge [22,23].

### 4.4. Integrative Perspective and Multi-Omics Relevance

Our integrative analyses suggest that metabolic exhaustion (ATP depletion) and microbial dysbiosis—rather than conventional lipids—were most tightly associated with prolonged hospitalisation [24,25]. This accords with the direction of travel in multi-omics CVD research, which seeks composite fingerprints across lipid, metabolomic, and microbial layers to refine risk stratification and discover actionable targets. Recent overviews in Atherosclerosis detail how multi-omics can improve diagnostics and therapeutic tailoring in ASCVD beyond traditional risk factors, supporting our choice to evaluate multiple biological domains together [26,27].

Although multi-omics domains were assessed jointly, the associations identified remain correlational and do not establish mechanistic or directional relationships between lipid, metabolomic, and microbiome alterations. The retrospective design and absence of time-resolved omic layers further limit causal inference. These findings should therefore be interpreted as associative and hypothesis-generating.

Cross-domain relationships identified in this study rely on associative modelling rather than advanced integrative inference, and therefore should be interpreted with caution.

Adjustment for pharmacotherapy revealed modest attenuation of the associations between prolonged hospitalisation, dysbiosis, and mitochondrial energy-related metabolites. This attenuation is consistent with the known effects of antibiotic exposure on *Enterococcus* enrichment and microbiome diversity, and with the influence of statins on lipid and amino acid metabolism. However, the persistence of statistically significant associations after adjustment supports the partial independence of these biological signatures. Given the dynamic, indication-driven nature of inpatient medication use, residual confounding is likely, and larger prospective cohorts with structured medication metadata will be required to determine causality.

The within-group temporal analyses lend additional support to the biological plausibility of our findings: energy-related metabolite depletion and increasing reliance on ketones, accompanied by declining microbial diversity and rising dysbiosis over the admission course in long-term patients. While these trajectories reinforce the main conclusions, they remain exploratory due to sparse and uneven sampling, dynamic inpatient exposures, and limited power. Larger prospective cohorts with predefined sampling schedules will be necessary to confirm time-dependent patterns and disentangle treatment- and disease-driven effects.

### 4.5. Clinical Implications

Practically, our data argue for (i) embedding serial metabolomic/microbiome assessments during extended admissions to anticipate clinical deterioration and (ii) interpreting inpatient lipid panels with caution, given short-term treatment and catabolic effects. Guideline-concordant Lp(a) assessment remains warranted for long-term cardiovascular risk management, even if acute in-hospital lipid snapshots are variable [28].

Multivariate models included major comorbidities as covariates to mitigate clinical confounding, although residual effects remain possible.

The alignment between cross-sectional contrasts and temporal trajectories supports biological plausibility while remaining hypothesis-generating due to limited sampling density.

### 4.6. Strengths and Limitations

Our study’s strengths include its retrospective design, harmonised sampling (baseline, every five days, discharge), and cross-domain profiling.

The application of structured LC-MS quality control with pooled QC samples, internal standardisation, and drift monitoring supports the analytical robustness of the metabolomic results.

Additionally, the lack of pre-admission baseline measurements and post-discharge follow-up limits temporal interpretation and prevents determination of whether the molecular profiles observed reflect pre-existing patient characteristics or biologically emergent features of prolonged hospitalisation. Furthermore, the biochemical subsample for lipid sub-analyses was modest, which may attenuate statistical power and introduce imprecision in group-level lipid estimates.

Although demographic and clinical differences were clearly presented, the sample size within specific clinical subgroups (e.g., diabetes, atrial fibrillation, sex-based strata) was insufficient to support statistically powered subgroup analyses. Attempting such comparisons in the present dataset would likely yield unstable estimates and risk overinterpretation. Larger, prospectively designed cohorts are required to determine whether the multi-omic signatures observed vary meaningfully across these clinical categories.

These limitations necessitate cautious interpretation and highlight the need for larger, prospective studies with structured longitudinal sampling. Limitations include small biochemical subsamples for certain lipid measures and the absence of pre-admission baselines, both of which may attenuate between-group contrasts in classical lipids and inflate uncertainty around effect sizes. These issues are common in in-hospital studies and should motivate larger, multi-centre replications with pre- and post-discharge follow-up.

In particular, the small biochemical subsample available for detailed lipid analyses attenuates statistical power and may introduce imprecision in effect estimation. Consequently, quantitative differences in lipid parameters should be interpreted with caution and validated in larger cohorts with complete longitudinal sampling. The very small biochemical subsamples for selected lipid parameters (particularly LDL-C and triglycerides) markedly limit statistical power and increase the risk of type II error. As a result, lipid-related trends should not be considered confirmatory. Post hoc power estimation is not meaningful under such sparse sampling; instead, estimation with wide confidence intervals and sensitivity checks were prioritised, and we explicitly avoided firm claims for underpowered comparisons. Future studies should be prospectively powered with complete lipid panels across serial time points to enable precise effect estimation.

Another limitation of the present work is the absence of pre-hospitalization biological controls, which restricts the ability to determine whether the observed lipid, metabolomic, and microbiome alterations represent pre-existing patient phenotypes or changes acquired during inpatient care. Moreover, the study lacks validation across broader external cohorts or multi-centre datasets, which may limit generalisability and reduce ecological validity. Future research should incorporate pre-admission sampling and include external cohorts to better characterise temporal trajectories and ensure reproducibility across heterogeneous clinical settings.

This retrospective, single-centre study has several limitations that qualify inference and external validity. It is also important to consider the potential influence of time-varying confounding factors inherent to prolonged inpatient care. Medication titration (including diuretics, beta-blockers, and statins), antimicrobial administration, dietary modification dictated by hospital menus, reduced mobility, and fluctuating inflammatory burden may each contribute to the observed metabolic and microbiome signatures. These exposures are heterogeneous and often evolve dynamically during admission, complicating the attribution of molecular changes to hospitalization duration alone. Although we harmonised sampling intervals to mitigate some of these effects, prospective studies with detailed tracking of medication, nutrition, physical activity, and antimicrobial exposure are warranted to better delineate their contributions.

First, although the overall cohort was modest (*n* = 92), assay-level completeness was uneven—particularly for some lipid determinations—reducing power for between-group contrasts and subgroup analyses (e.g., sex, diabetes). Second, as an observational design without pre-admission baselines or post-discharge follow-up, the study cannot establish causality; residual and time-varying confounding (medication intensification, dietary changes, antimicrobial exposure, intercurrent illness) likely influenced lipid, metabolomic and microbiome readouts despite harmonised sampling every five days. Third, laboratory and bioinformatic sources of bias may persist (batch effects in LC-MS, compositional constraints in 16S data, platform-specific dysbiosis indices), even with standardised protocols. Fourth, selection factors—such as the ability to provide stool samples and remain inpatient long enough for serial testing—introduce potential selection and survivorship biases.

Not all detected metabolites were confirmed by MS/MS fragmentation due to low abundance and instrument duty cycle constraints. Therefore, some identifications should be regarded as putative, reflecting Level 2 confidence according to accepted metabolomics reporting criteria.

Despite statistical control for repeated measures and key covariates, broader residual confounding remains possible, reflecting the heterogeneous and complex inpatient environment.

Dynamic inpatient medication adjustments limit our ability to isolate drug effects from disease-related signatures.

Finally, the study was conducted in a specialised cardiovascular department of a university hospital, which may limit generalisability to community hospitals, short-stay cardiology wards, non-cardiac populations, intensive care settings, or health systems with different care pathways and dietary/regional microbiome backgrounds. Accordingly, these findings should be viewed as hypothesis-generating, pending validation in larger, multi-centre cohorts with structured baseline and longitudinal follow-up.

External replication will be required to validate dysbiosis and ATP depletion as predictors of prolonged hospitalisation and to determine their clinical applicability.

It is important to acknowledge that, due to the retrospective and observational nature of this study, no causal relationships can be inferred between prolonged hospitalisation and the molecular signatures described. Time-dependent inpatient exposures—including medication adjustments, dietary modification, antimicrobial administration, comorbidity progression, and variable functional status—may contribute to the observed alterations. Accordingly, the associations identified here should be interpreted as hypothesis-generating and require validation through prospective, longitudinal investigations.

### 4.7. Future Directions

Our findings support trial-ready hypotheses: (a) targeted metabolic support (e.g., ketone pathway modulation) in patients exhibiting ATP-depleted signatures; (b) microbiome-directed interventions to mitigate dysbiosis during long stays; and (c) development of multi-omics predictive scores to guide step-down planning and readmission prevention, as advocated by recent translational roadmaps.

Future studies should prioritise complete lipid panel acquisition across serial timepoints to improve statistical resolution and enable more precise modelling of in-hospital lipid dynamics. Also, they should include sufficient representation of clinically relevant subgroups (e.g., diabetic vs. non-diabetic patients, sex-specific analyses) to determine whether molecular signatures associated with prolonged hospitalisation exhibit phenotype-specific patterns.

Prospective, multi-centre cohorts with pre-admission sampling and external validation arms will be crucial to confirm the reproducibility of the observed signatures and to improve the translational potential of multi-omics profiling in extended cardiac hospitalisation. We should discuss the results and how they can be interpreted from the perspective of previous studies and of the working hypotheses. The findings and their implications should be discussed in the broadest context possible. Future research directions may also be highlighted.

Regarding these findings, our future work should incorporate external validation cohorts to confirm the predictive performance of dysbiosis indices and bioenergetic exhaustion markers and to evaluate their value for risk stratification in real-world inpatient settings.

Future longitudinal studies should incorporate high-resolution metadata on medication regimens, nutritional content, mobility level, and antimicrobial exposure to quantify their impact on molecular profiles and to disentangle patient-related from hospitalization-acquired signatures.

## 5. Conclusions

In this retrospective cohort of cardiac inpatients, an integrated, serial assessment across lipids, plasma metabolomics, and the gut microbiome revealed a coherent biological signature associated with prolonged hospitalisation. While conventional lipid measures showed limited and sample-restricted differences between groups, long-term stays were characterised by an energy-depleted metabolic state (lower ATP/phosphocreatine indices, greater ketone reliance) and microbiome disruption (reduced α-diversity and higher dysbiosis index). In multivariate models, dysbiosis and ATP depletion—rather than classical lipids—emerged as the most informative correlates of extended length of stay, underscoring the value of multi-omics profiling for in-hospital risk stratification.

Notably, the reliability of lipid-related trends is constrained by the very small biochemical subsample size for LDL cholesterol and triglycerides, and these observations should therefore be regarded as exploratory.

These findings support embedding serial metabolomic and microbiome readouts into clinical pathways for patients with complex cardiovascular disease and motivate targeted, testable interventions to restore energetic balance and microbial resilience.

These predictive signatures remain hypothesis-generating and require external validation before consideration in clinical decision-support pathways

Given the retrospective design of this study, these findings reflect associations rather than causative mechanisms, and caution is warranted when extrapolating temporal or mechanistic inferences. Prospective studies with structured baseline and post-discharge follow-up will be essential to determine whether the molecular alterations observed actively contribute to prolonged hospitalisation or represent downstream consequences of advanced clinical severity.

Lipid differences between groups cannot be stated firmly given the very small subsamples and associated type II error risk; larger, adequately powered cohorts are required. The multi-omics relationships identified here are correlational and require prospective, temporally resolved profiling to determine whether these domains interact mechanistically during prolonged hospitalisation. Our multi-omics observations provide preliminary, correlational insight into biological alterations associated with prolonged hospitalisation. Deeper statistical integration and validation in larger cohorts will be required to define mechanistic cross-domain interactions.

Validation in larger, multi-centre cohorts with structured baseline and post-discharge follow-up is warranted.

## Figures and Tables

**Table 1:** Demographic and Clinical Characteristics of Study Groups.

Characteristic	Short–Intermediate Stay (3–24 Days, *n* = 41)	Long-Term Stay (≥25 Days, *n* = 51)
Age (years, range)	49–90	45–93
Age (median)	70	71
Age (mean)	68.6	71.8
Insufficiency heart failure (mentions)	33	48
Ischaemic heart disease (mentions)	10	6
Hypertension (mentions)	12	9
Diabetes mellitus (mentions)	4	6
Atrial fibrillation (mentions)	3	5
Stroke (mentions)	3	1
Obesity (mentions)	1	1

**Table 2:** Lipid profile of cardiac patients by duration of hospitalisation.

Variable	Control (3–24 Days)	Long-Term (≥25 Days)
Total cholesterol	137.6 ± 7.3 mg/dL (*n* = 3)	142.7 ± 14.4 mg/dL (*n* = 3)
LDL cholesterol	113.3 ± 8.6 mg/dL (*n* = 3)	66.4 ± 25.2 mg/dL (*n* = 2)
HDL cholesterol	57.1 mg/dL (*n* = 1)	61.6 mg/dL (*n* = 1)
Triglycerides	158.6 ± 113.0 mg/dL (*n* = 3)	62.8 mg/dL (*n* = 1)

**Table 3:** Summary of metabolomic differences between control and long-term hospitalisation groups.

Metabolite Category	Control (3–24 Days)	Long-Term (≥25 Days)	Direction of Change
ATP	Reference range	↓ Reduced	↓
Phosphocreatine ratio	Reference range	↓ Reduced	↓
Lactate	Normal	↑ Elevated	↑
Ketone bodies	Low	↑ Increased	↑
BCAA (Val, Leu, Ile)	Normal	↓ Reduced	↓
Aromatic AA (Phe, Tyr, Trp)	Normal	↑ Increased	↑
Acylcarnitines	Normal	↑ Altered	↑
Phospholipids	Normal	↓ Altered	±

**Table 4:** Gut microbiome characteristics by hospitalisation duration.

Parameter	Control (3–24 Days)	Long-Term (≥25 Days)	Direction of Change
Shannon diversity index	Reference/normal	↓ Reduced	↓
Simpson index	Reference/normal	↓ Reduced	↓
β-diversity (Bray–Curtis)	Within normal variation	Distinct clustering	—
Dysbiosis index	Low–moderate	↑ Increased	↑
Firmicutes (%)	Normal abundance	↓ Reduced	↓
Bacteroidetes (%)	Normal abundance	↓ Reduced	↓
Proteobacteria (%)	Low abundance	↑ Increased	↑
*Enterococcus* spp.	Absent/rare	↑ Present	↑
*Candida* spp.	Rare	↑ Overrepresented	↑

**Table 5:** Integrative correlations between biological markers and clinical outcomes.

Variable	Test Applied	Association with Hospital Stay	Association with Severity Score
LDL cholesterol	*t*-test	↓ *p* < 0.05	ns
Triglycerides	*t*-test	↓ *p* < 0.05	ns
HDL cholesterol	*t*-test	ns	trend ↑ (ns)
ATP, phosphocreatine	RM-ANOVA/Spearman	↓ *p* < 0.01	ρ = –0.44, *p* < 0.01
Ketone bodies	RM-ANOVA/Spearman	↑ *p* < 0.05	ρ = 0.40, *p* < 0.05
BCAA (Val, Leu, Ile)	Mann–Whitney U	↓ *p* < 0.05	ns
Aromatic AA (Phe, Tyr, Trp)	Mann–Whitney U	↑ *p* < 0.05	ρ = 0.35, *p* < 0.05
Dysbiosis index	Mann–Whitney U	↑ *p* < 0.01	ρ = 0.46, *p* < 0.01
Shannon diversity	Mann–Whitney U	↓ *p* < 0.01	ρ = –0.37, *p* < 0.05

## Data Availability

The original contributions presented in this study are included in the article/Supplementary Material. Further inquiries can be directed to the corresponding author.

## Supplementary Materials

The following supporting information can be downloaded at: https://www.mdpi.com/article/10.3390/diagnostics15222874/s1, Table S1: Evolution of metabolic and microbial indices at baseline, mid, and discharge; Table S2: Long-term cardiac patient medication profile; Figure S1: Metabolic and Microbiome Adaptations.
