# Supplementary figures and images for: Lipid, Metabolomic and Gut Microbiome Profiles in Long-Term-Hospitalized Cardiac Patients—An Observational and Retrospective Study
Source: Diagnostics (Basel). 2025 Nov 13;15(22):2874. doi: 10.3390/diagnostics15222874 (PMC12651827; doi:10.3390/diagnostics15222874)

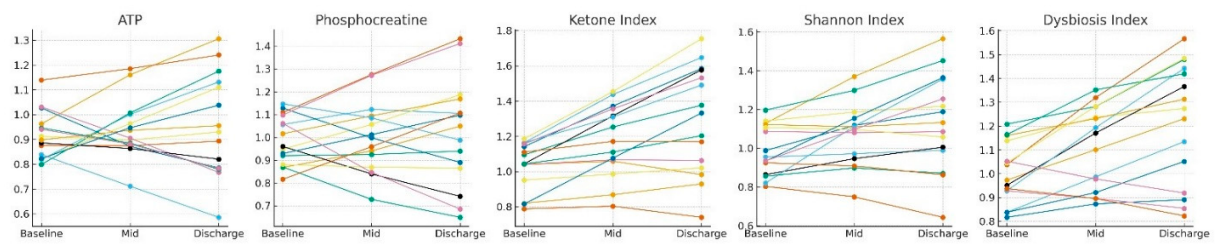

**Figure S1.** Metabolic and Microbiome Adaptations.

Supplement: Supplementary file 1 [file diagnostics-15-02874-s001.zip › Figure S1.pdf]
